# An audit into the use of psychotropic medications in patients with autism spectrum disorder in a high security psychiatric hospital

**DOI:** 10.1192/bjo.2021.222

**Published:** 2021-06-18

**Authors:** Elliott Carthy, David Murphy

**Affiliations:** Broadmoor Hospital, West London NHS Trust

## Abstract

**Aims:**

Autism Spectrum Disorder (ASD) is a common neurodevelopmental disorder associated with difficulties in social communication and language development, preoccupations, a need for routine, sensory sensitivities and emotional dysregulation. People with autism who have violently offended may be prescribed psychotropic medications to treat comorbidities, or off-license to manage aggressive or challenging behaviours. However, the evidence base for their use remains scarce.

**Method:**

This was a retrospective audit at Broadmoor Hospital, a high security psychiatric hospital in the United Kingdom, into the safe and appropriate prescribing of psychotropic medicines in those with an ASD compared to guidance from the National Institute for Health and Care Excellence (CG142): “Autism spectrum disorder in adults: diagnosis and management”. This first cycle was undertaken during May and June 2020 and included all patients with a confirmed or equivocal diagnosis of ASD in the preceding five years.

**Result:**

A total of 22 participants were included in this study. Of these, 17 participants had a confirmed diagnosis of ASD and five participants had a suspected diagnosis of ASD, but without formal confirmation with neurocognitive testing. A total of 13 (76.5%) participants with confirmed ASD were prescribed antipsychotic medication, nine of whom had an established comorbid mental disorder with psychotic symptoms. Of the remaining four, three had a diagnosis of a personality disorder. Three participants in this study had a confirmed diagnosis of ASD without any additional comorbid mental health diagnoses. No patients were prescribed psychotropic medicines for the core symptoms of ASD. The specific documentation of off-license use of antipsychotic medicines in those without a diagnosis of a psychotic disorder was poor. This was not recorded in any such participant in the preceding 12 months.

**Conclusion:**

This audit highlighted that dual diagnoses of ASD alongside non-affective psychosis and personality disorder are over-represented in this high security setting. The NICE clinical guidelines CG142 guidelines state that “antipsychotic medications should only be used for behaviour that challenges if …. the risk to the person or others is very severe”. By definition, all patients admitted to high security are deemed to be a grave and imminent risk to the public. Psychotropic medicines may therefore be clinically indicated at a much earlier stage than in community patients, instigated alongside appropriate psychosocial interventions and treatment of comorbid conditions. It may be that catered guidelines need to be formulated to support the safe and appropriate prescribing of psychotropic medicine in forensic settings.

